# Transcriptomic analysis of the inhibition mechanisms against *Pseudomonas plecoglossicida* by antibacterial aptamer B4

**DOI:** 10.3389/fvets.2024.1511234

**Published:** 2024-12-24

**Authors:** Ying Tan, Xiaojun Lin, Lixing Huang, Qingpi Yan, Jiaen Wang, Qibiao Weng, Yuwei Zhengzhang, Yiran Chen, Ying Ma, Jiang Zheng

**Affiliations:** ^1^State Key Laboratory of Mariculture Breeding, Engineering Research Centre of the Modern Technology for Eel Industry, Ministry of Education, Fisheries College of Jimei University, Xiamen, China; ^2^National Research and Development Center for Eel Processing Technology, Key Laboratory of Eel Aquaculture and Processing of Fujian Province, Fujian Provincial Engineering Research Center for Eel Processing Enterprise, Changle Juquan Food Co. Ltd., Fuzhou, China; ^3^Institute of Traditional Chinese Medicine, Fujian University of Traditional Chinese Medicine, Fuzhou, China; ^4^School of Life Sciences, Xiamen University, Xiamen, China

**Keywords:** *Pseudomonas plecoglossicida*, aptamers, antibacterial, transcriptomics, fish disease, RNA-seq

## Abstract

*Pseudomonas plecoglossicida* is a common bacterial pathogen in aquaculture, often leading to visceral white spot disease in large yellow croakers (*Pseudosciaena crocea*). Previous studies have found that certain aptamers show an efficient antibacterial effect against this pathogen. In this study, we analyzed the transcriptome of *P. plecoglossicida* to get insights into the antibacterial and inhibitions mechanisms following exposure to the aptamer B4. The results showed seven differentially expressed genes (DEGs) associated with the antibacterial effect of the aptamer, namely *sad* gene encoding aldehyde dehydrogenase, the *paaB* gene of phenylacetyl coenzyme A cyclooxygenase, the *metN1* gene of ABC transporter proteins, two transposase genes with different positions but identical sequences involved in cutting and splicing DNA sequences, and two hypothetical protein genes with unknown functions. Gene Ontology (GO) analysis showed that the DEGs were mainly involved in DNA-mediated translocation, phenylacetic acid catabolism, growth hormone catabolism, polyamine transporter ATPase activity, betaine aldehyde dehydrogenase activity, ABC transporter protein complex, and other related pathways. Kyoto Encyclopedia of Genes and Genomes (KEGG) analysis showed that the metabolic pathway of niacin and niacinamide mediated through the *sad* gene was the most significant and relevant, followed by the metabolism of phenylalanine, alanine, aspartic acid and glutamic acid. Real-time quantitative PCR validation showed that the changes in the DEGs were consistent with the transcriptome analysis. These results suggest that the antibacterial aptamer B4 may inhibit *P. plecoglossicida* by blocking the synthesis of essential nucleic acids and proteins through the modulation of these DEGs and inhibiting their metabolic pathways.

## Introduction

*Pseudomonas plecoglossicida* is a Gram-negative bacteria commonly found in water, soil, animals and plants. It is an important pathogen in aquaculture, often causing visceral white spot disease in large yellow croakers (*Pseudosciaena crocea*) and severe economic losses in marine aquaculture (1–4). Currently, antibiotics are primarily used to treat affected fish and prevent the spread of the disease. However, the long-term overuse of antibiotics not only pollutes the environment but also leads to the development of drug-resistant bacteria (5). In addition, antibiotics also significantly impact the fish’s gut microbiota, further compromising the survival of the fish (6). As a result, it is essential to explore more effective and efficient methods of controlling disease occurrence and outbreaks.

Aptamers are artificial and single-stranded oligonucleotide molecules that bind a specific target molecule with a high affinity and specificity (7). Compared to antibody proteins, aptamers have many advantages including easy modification and synthesis, wider target range, better stability, easy transportation and storage, and high affinity and specificity (8, 9). Nowadays, aptamers are widely utilized in various fields such as disease diagnosis and treatment, analysis and detection, and targeted therapy (10–12).

We have obtained antibacterial aptamers that can inhibit *P. plecoglossicida* through previous studies and investigated their antibacterial properties (13). The study showed that the antibacterial rate of aptamer B4 against *P. plecoglossicida* was 62.40 ± 4.17%, and its binding protein was the 26-kDa ribosomal protein L2. The aptamer is likely to interfere with protein synthesis by interacting with ribosomal protein, thus inhibiting the growth of *P. plecoglossicida*. However, the mechanism of the antibacterial aptamer in terms of gene and transcriptome remains unclear.

In this paper, we used transcriptomics to study the antibacterial mechanism of aptamers against *P. plecoglossicida* through RNA sequencing, differential gene screening, and gene enrichment and predicted functional annotation using Gene Ontology (GO) and Kyoto Encyclopedia of Genes and Genomes (KEGG). The outcomes of this research will lay the foundation for the research and development of antibacterial aptamers and mitigate the risks of disease outbreaks in fish farms and aquacultures.

## Materials and methods

### Microorganisms, aptamers and primers

*Pseudomonas plecoglossicida* was obtained from the Laboratory of Pathogenic Microorganisms, Jimei University. The bacteria were cultured in LB (Luria-Bertani) broth at 18°C and 100 rpm in a shaking incubator (Changzhou National Test Equipment Research Institute, Changzhou, China) for 12 h.

The aptamer B4 obtained from a previous study (13) showed a better inhibitory effect against the growth of *P. plecoglossicida*. Aptamer B4 sequence: 5′-TCAGTCGCTTCGCCGTCTCCTTCCGTACCGCCTGCGGTGGATCGTATTGTGGAACGTGGCACAAGAGGGAGACCCCAGAGGG-3′. The aptamer and primers used in the paper were purchased from Shanghai Sangon Bioengineering Co. Ltd. (Shanghai, China).

### Sample treatment and RNA sequencing

The 20× binding buffer for aptamer binding was composed of 0.9 mol/L NaCl, 0.5 mol/L KCl, 0.4 mol/L Tris–HCl, 0.1 mol/L MgCl_2_ with pH 7.4, which was sterilized at 121°C for 25 min, and diluted to 2× or 1× working solution with sterile ultrapure water.

Cultures of *P. plecoglossicida* in broth were centrifuged at 6000 rpm for 5 min, the supernatant discarded, and the precipitate washed twice with 1× binding buffer solution, and then diluted to 10^5^ CFU/mL bacterial suspension with 1× binding buffer solution. Five milliliters of 1 μM aptamer B4 were heated in a heating block at 95°C for 5 min and then placed in an ice bath for 10 min. In the experimental group (EG1, EG2, and EG3), 5 mL of 1 μM treated aptamer B4 was mixed with 5 mL of 10^5^ CFU/mL suspension of *P. plecoglossicida*, while for the control group (BG1, BG2, and BG3), the bacterial suspension was mixed with only 5 mL 1× binding buffer instead of the aptamer solution. Three replicates were set up for each experimental and control group. All the groups were incubated at 100 rpm and 18°C for 1 h. Each sample was centrifuged at 13000 rpm for 10 min to collect the bacterial pellet and the tubes were placed into 50 mL conical tubes to prevent freezing and cracking. The conical tubes were placed in liquid nitrogen for 5 min and then removed. The small centrifuge tubes containing the bacterial pellets were retrieved, then sealed with a sealing film, and stored at −80°C before testing.

Three samples from each of the experimental and the control groups were stored in dry ice and sent to Guangzhou Kidio Biotechnology (GKB) Co. Ltd. for RNA extraction followed by RNA sequencing.

RNA extraction, sequencing library construction, sequencing, and raw sequencing data filtering were all completed by GKB Co. Ltd. First, the total RNA of the samples was extracted, and cDNA was constructed after rRNA removal and fragmentation. Then PCR amplification of the cDNA was performed to construct the corresponding sequencing library. Finally, the sequencing was carried out on the Illumina platform of GKB Co. Ltd. to obtain the raw sequencing data. The raw sequencing data were filtered using the quality control software fastp (14) of GKB Co. Ltd., to remove the reads containing joints, the reads of all A bases, the reads with N proportion more than 10%, and the reads with the bases of Q ≤ 20 accounting for more than 50%., Finally the clean data were obtained after the raw data were filtered.

### Screening and enrichment analysis of differentia gene expression

Differentia gene expression (DEGs) was screened through the data analysis platform of GKB Co. Ltd. The screening criteria were |log_2_FC| ≥ 1 and *p* ≤ 0.05, that is, genes that met the difference multiple greater than 2 times and *p* ≤ 0.05 after correction were considered DEGs.

After obtaining the DEGs, the GO (Gene Ontology) and KEGG (Kyoto Encyclopedia of Genes and Genomes) databases were used to perform functional annotations and identify the associated metabolic pathways based on the R language software package clusterProfiler of GKB Co. Ltd. The threshold was set as *p* ≤ 0.05 for significant enrichment to identify processes and pathways associated with repression.

### Validation of differentia gene expression by RT-qPCR

**Figure 1:**
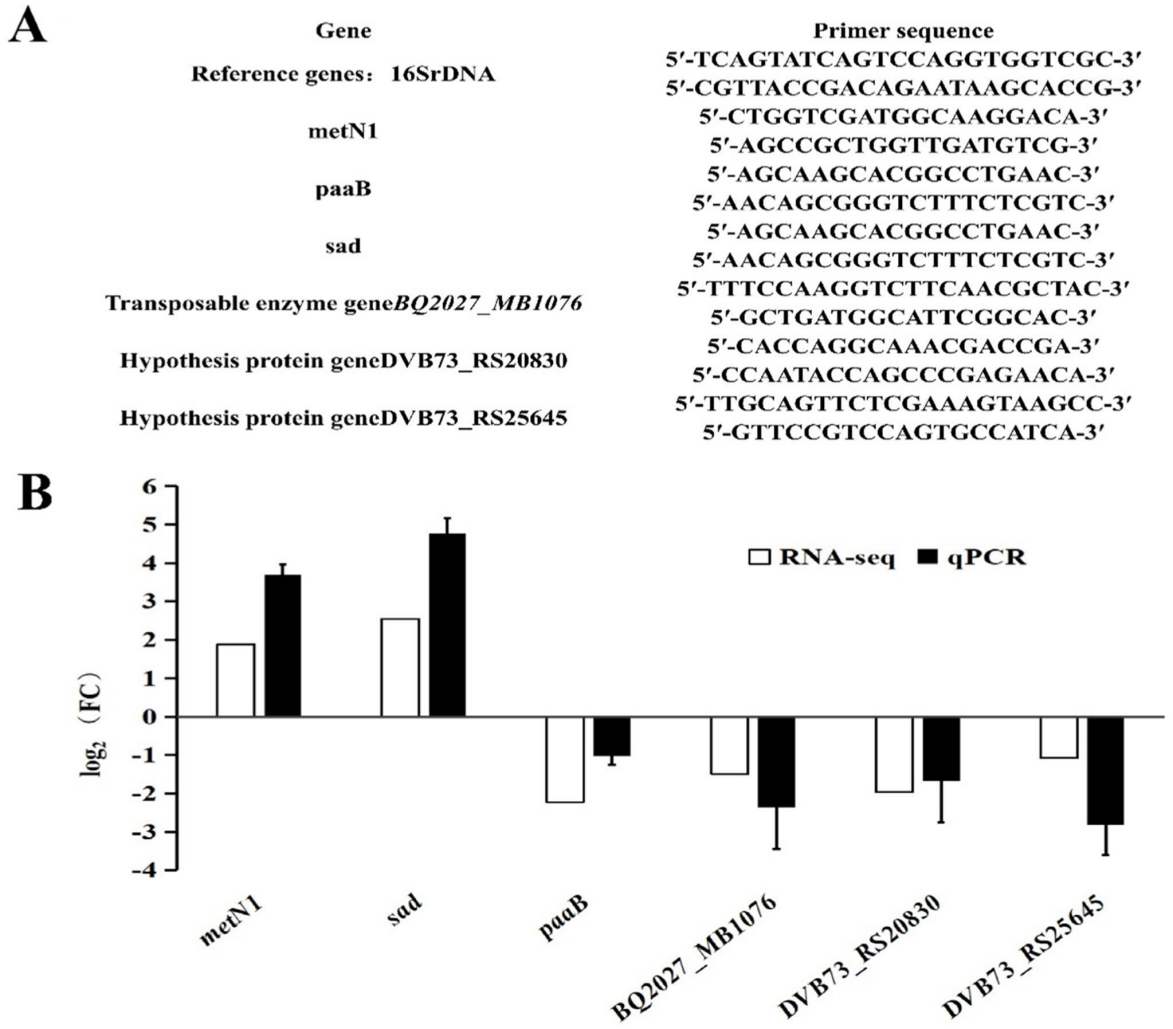
Verification of differentia gene expression by qPCR. **(A)** qPCR primers. **(B)** Comparison of transcriptome data and qPCR results.

Changes in differentia gene expression were validated by reverse transcription quantitative real-time PCR (RT-qPCR). First, Six mRNA samples extracted by GKB Co. Ltd. were used as templates to form cDNA using the Evo M-MLV reverse transcription kit (Accurate Biology Co. Ltd., Changsha, China). The reverse transcription solution was composed of 10 μL RNase-free water, 5 μL mRNA, 4 μL reverse transcriptase and 1 μL gDNA Remover. The reverse transcription conditions were: 42°C for 15 min, then 85°C for 5 s. The cDNA obtained by reverse transcription was then used as the template for the SYBR Green I (Accurate Biology Co. Ltd., Changsha, China) based RT-qPCR. The 16S rRNA was used as the internal reference gene. The 10 μL reaction solution for qPCR was composed of 5 μL qPCR SuperMix, 4 μL ddH_2_O, 0.5 μL cDNA, 0.25 μL forward primer, 0.25 μL reverse primer (primer sequences listed in Figure 1A). The amplification parameters of qPCR were as follows: pre-denaturation at 94°C for 5 min, followed by 40 cycles of denaturation at 94°C for 30 s, annealing at 60°C for 30 s, extension at 72°C for 3 min, and final extension at 72°C for 10 min.

### Data access

The RNA-seq data has been uploaded and stored in the Genome Sequence Archive (GSA) database, which is available through the GSA website^1^ under registration number CRA019590.

## Results

### Screening and validation of differentially expressed genes

**Figure 2:**
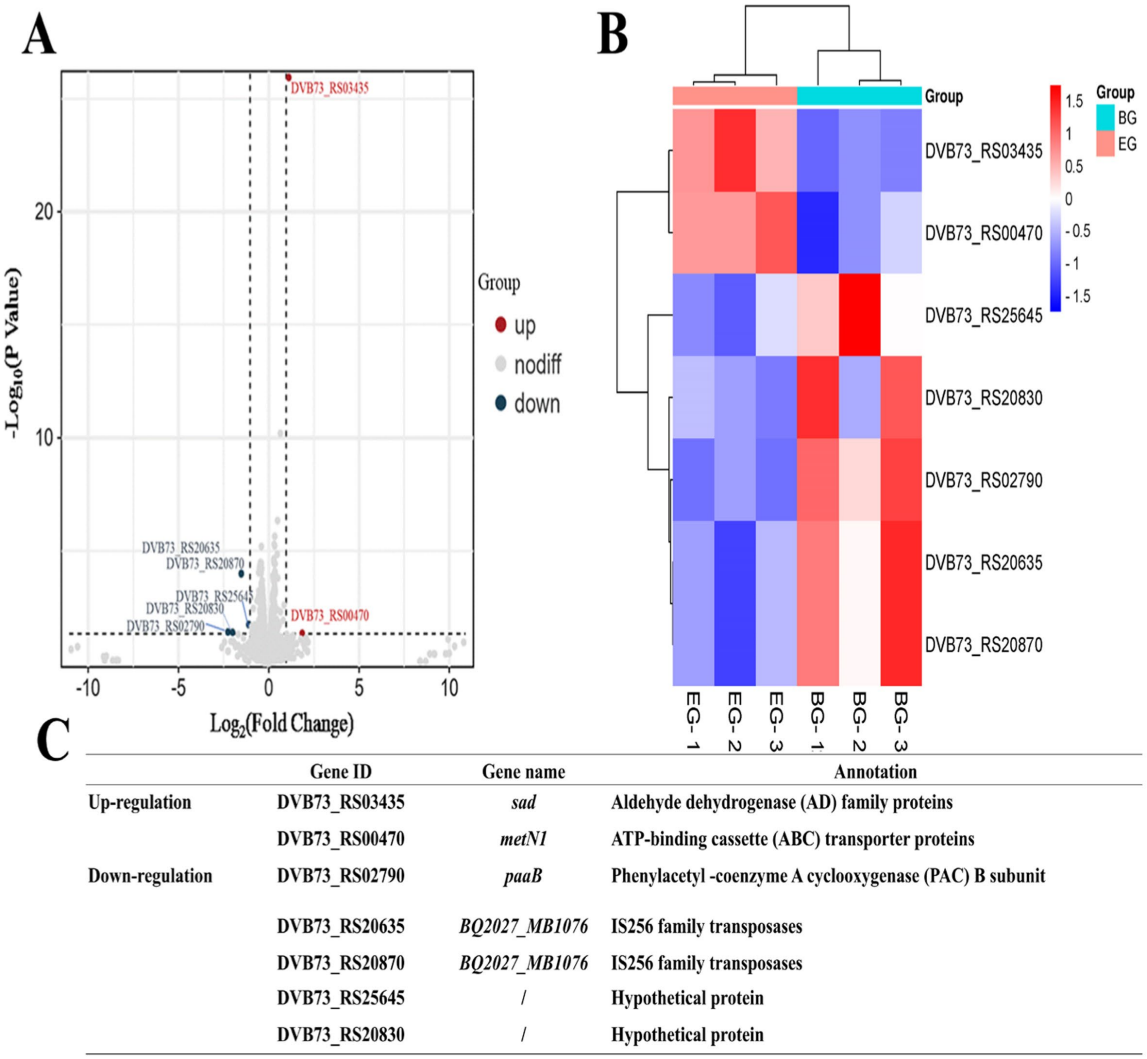
Volcano map **(A)**, heat map **(B)**, name and annotations **(C)** of differentia gene expression.

Seven differentially expressed genes were identified, two up-regulated and five down-regulated, as shown in Figures 2A,B. The two up-regulated genes were associated with the aldehyde dehydrogenase (AD) family proteins and ATP-binding cassette (ABC) transporter proteins, while the five down-regulated genes were connected to the phenylacetyl-coenzyme A cyclooxygenase (PAC) subunit B, IS256 family transposases, and two hypothetical proteins with unknown functions (Figure 2C). Both the transposase genes are the typical transposons of *P. plecoglossicida* and have identical sequences, with the only difference in their location leading to their different gene IDs.

### GO analysis of differentially expressed genes

**Figure 3:**
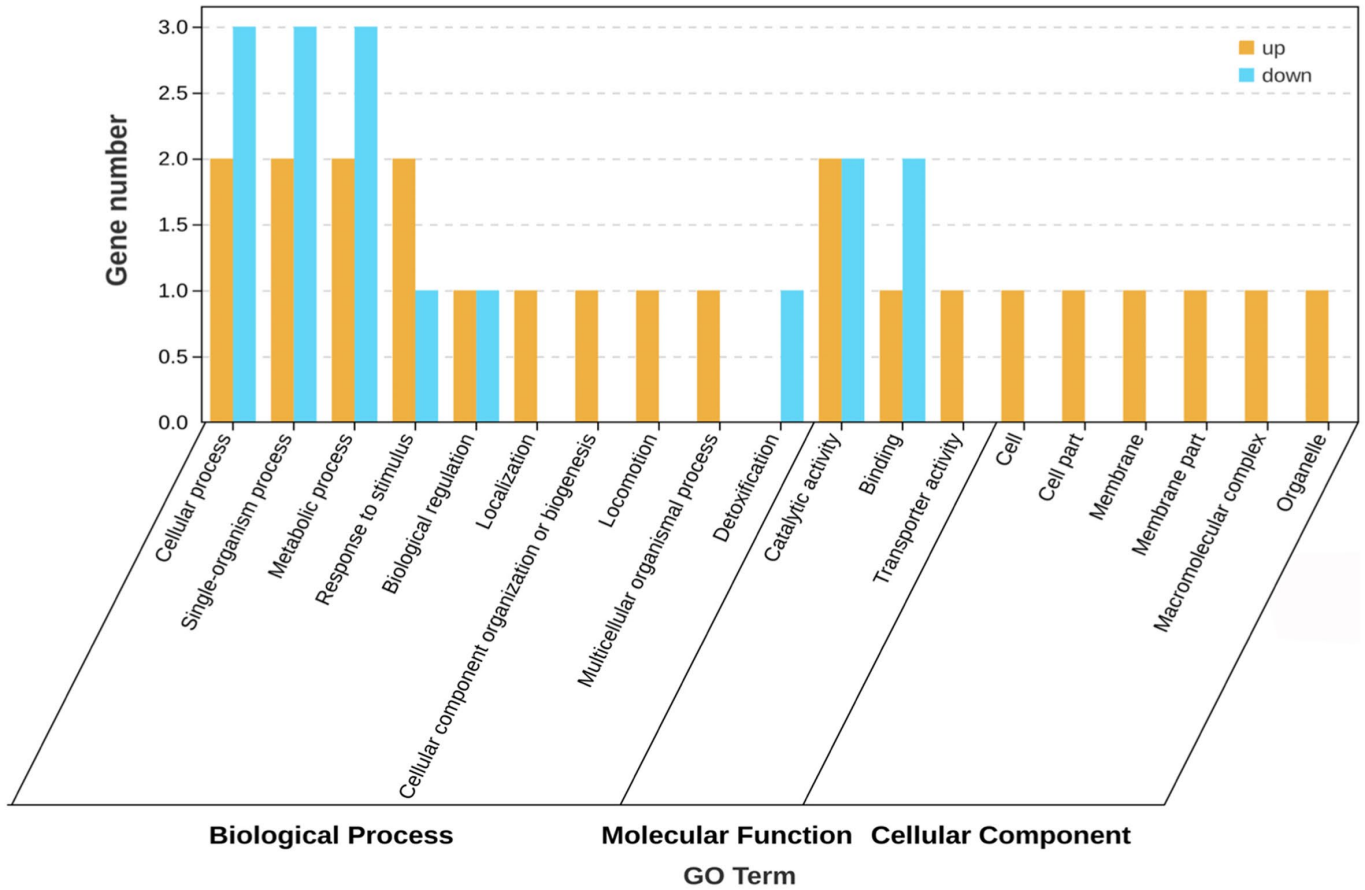
GO functional annotation of differentia gene expression.

The GO database helps identify the association of genes with biological processes, molecular functions, and cellular components. GO enrichment analysis was performed on the five differentially expressed genes (*sad*, *metN1*, *paaB* and two transposase genes) with the known functions, and the results are shown in Figure 3. These genes are involved in various biological processes such as cellular, metabolic, and single or multicellular-organism processes, response to stimulus, biological regulation, cellular component biological organization or biogenesis, localization, locomotion, and detoxification. The molecular function of these differentially expressed genes is mainly associated with catalytic, binding, and transport activities. They are also involved in cellular components, including associations with cell and membrane parts, macromolecular complexes, and organelles. The five differentially expressed genes showed various connections to biological processes, molecular functions, and cellular components. In biological processes, they were mainly associated with DNA-mediated translocation, phenylacetic acid catabolic processes, and growth hormone catabolic processes, or molecular functions connected to polyamine-translocating ATPase activity and betaine aldehyde dehydrogenase activity, and cell component activities related to ABC transporter protein complexes.

### KEGG analysis of differentially expressed genes

**Figure 4:**
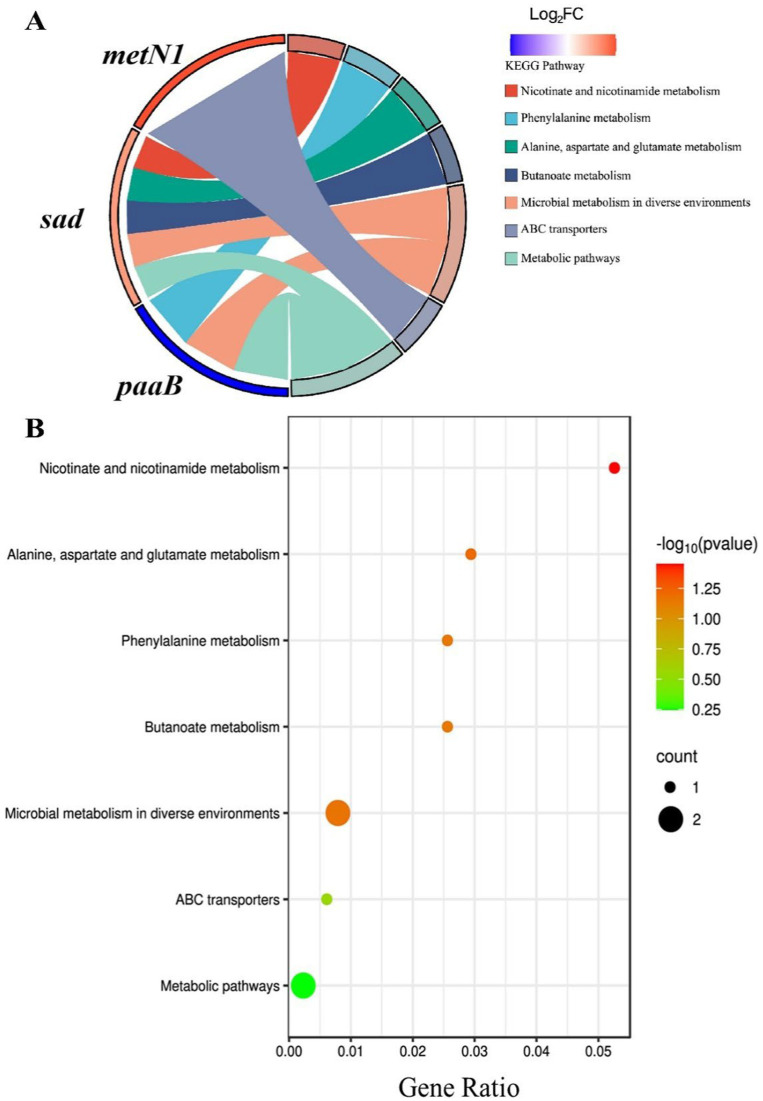
Chord diagram **(A)** and bubble diagram **(B)** of KEGG analysis.

KEGG pathway analysis of the five differentially expressed genes (*metN1*, *sad*, *paaB*, and two transposase genes) with the known functions indicated that only *metN1*, *sad*, and *paaB* (DVB73_RS00470, DVB73_RS03435, and DVB73_RS02790, respectively) were involved in the seven identified pathways, while the two transposase genes did not show any involvement (Figure 4A). The up-regulated gene *sad* involved five pathways (nicotinic acid and nicotinamide metabolism pathway; alanine, aspartate and glutamate metabolism; metabolism of methyl butyrate; microbial metabolism in different environments; and microbial metabolism pathway), while *metN1*, which was also up-regulated, was only involved in the ABC transporters pathway. The down-regulated gene *paaB* was involved in three pathways (phenylalanine metabolism; microbial metabolism in different environments; and microbial metabolic pathways).

The bubble diagram of Figure 4B showed that the seven pathways were connected to these three genes (*metN1*, *sad*, and *paaB*). Among them, four pathways involved with *sad* gene (nicotinic acid and nicotinamide metabolism pathway, metabolism of alanine, aspartic acid and glutamic acid, metabolism of methyl butyrate, metabolism of microorganisms in different environments) and two pathways involved with *paaB* gene (metabolism of phenylalanine, metabolism of microorganisms in different environments) were all red in color, indicating significant and high correlations. The nicotinic acid and nicotinamide metabolism pathways were the most significant and highly correlated pathways, while the ABC transporters and the microbial metabolism pathways related to the *metN1* gene were less significant. These results indicate that among the three genes, *sad* and *paaB* genes may play major roles following exposure to aptamers. The antibacterial aptamers may interfere with the metabolic pathways of nicotinic acid, nicotinamide and some amino acids in *P. plecoglossicida*, thus disrupting the adaptive metabolism of microorganisms in response to different environments and subsequently inhibiting the growth of the bacterium.

### qPCR validation of differentially expressed genes

Among the seven differentially expressed genes, two transposase genes were validated together using the same primers because of their identical sequences. The corresponding primer sequences are shown in Figure 1A and the validation results by qPCR are shown in Figure 1B. The qPCR results of the differentially expressed genes were consistent with the outcomes of the transcriptome analysis. The *metN1* and *sad* genes were up-regulated, while *paaB*, transposase genes (BQ2027_MB1076), and the two hypothetical protein genes (DVB73_RS20830, DVB73_RS25645) were down-regulated. However, the multifold expressions of the differentially expressed genes were not exactly the same as the transcriptome data, and that is most likely due to the differences in experimental and computational methods.

## Discussion

Transcriptomic analysis showed that the aptamer B4 impacts *P. plecoglossicida* mainly through modulating 7 genes, namely the *sad* gene encoding aldehyde dehydrogenase, *paaB* gene of phenylacetyl-CoA cyclocoxidase, *metN1* gene of ABC transporter, 2 transposase genes with different locations but identical sequences, and 2 hypothetical protein genes with unknown functions.

Aldehyde dehydrogenase is an essential enzyme in various biological processes such as biosynthesis, catabolism, and cellular detoxification (15). It plays an important role in cellular detoxification by degrading aldehyde intermediates produced as byproducts during metabolic processes in *Listeria monocytogenes*, *Vibrio paraholyticus* and *Lactobacillus reuteri* (16). In this study, we observed that the addition of the antibacterial aptamer led to the increased expression of the *sad* gene. This, in turn, affected four metabolic pathways involved in the metabolism of nicotinic acid and nicotinamide (NADH), alanine, aspartic acid, and glutamic acid. These findings indicate that *P. plecoglossicida* enhanced the degradation of aldehyde intermediates to counteract the effects of the aptamer. This disrupted the normal metabolic processes of NADH, alanine, aspartic acid, and glutamic acid and interfered with the microorganism’s adaptive metabolism in response to different environments, leading to the inhibition of bacterial growth.

Studies have shown that phenylacetyl-CoA cycoperoxidase has oxygenase activity and is a key enzyme in the catabolism process of Phe in bacteria, such as *Escherichia coli* and *P. plecoglossicida* (17). Through the action of the enzyme, phenylacetyl-CoA, an intermediate of Phe catabolism, can be epoxidized and further degraded by the tricarboxylic acid cycle (18). Our transcriptome analysis revealed that exposure to antibacterial aptamers resulted in the down-regulation of the *paaB* gene, which is involved in Phe metabolism and other metabolic pathways in *P. plecoglossicida*.

ABC transporter proteins are widely found in bacteria (19), which use the energy generated by ATP hydrolysis to achieve transmembrane transport of various substrate molecules (proteins, amino acids, bacterial secretions and metabolites, antibiotics, etc.) (20, 21). The outward transporter proteins of the ABC transporter proteins are present in all organisms, whereas the inward transporter proteins are present only in bacteria and plants (22, 23). In bacteria, the inward transporter proteins on the outer side of the cell membrane bind to the substrate first, and then to transmembrane transporter proteins, to complete the transport of the substrate (21, 24). This study showed that the addition of antibacterial aptamer significantly up-regulated the expression of the ABC protein gene *metN1*, indicating that the energy metabolism of the bacterium was significantly affected under the aptamer’s action, and it was speculated that the antibacterial aptamer might enter into bacterium by binding to the inward transporter proteins.

Transposase genes are associated with bacterial biofilm formation (25), which can greatly enhance bacterial drug resistance (26). The IS256 gene is an essential component of the complex transposon Tn4001, which can promote the formation of biofilms through transposition, insertion, and gene recombination of the *ica* operon (27). In certain species of *staphylococcus*, strains with high IS256 expression are more capable of biofilm formation (28). Moreover, the insertion of the transposon gene IS256 in *Pseudomonas aeruginosa* and increasing its density significantly increased the mutation rate of the bacterial genome and the development of antibiotic resistance (29). In the present study, the addition of the antibacterial aptamer led to the significant down-regulation of the transposase genes, followed by the inhibition of the growth of *P. plecoglossicida*. This observation suggests that the aptamer may affect the formation of biofilms and render them susceptible to antibacterials by inhibiting the expression of transposase genes, thereby reducing the capacity of bacteria to develop drug resistance and inhibiting their growth.

Hypothetical proteins (HPs) are a class of proteins with uncertain functions, constituting 30–40% of the genes in most bacterial genomes (30, 31). In *Pseudomonas* species, HPs often account for more than 36% of sequencing data, making the study of HPs very important for understanding bacteria (32). Pragati et al. (33) studied and annotated 96 biological functions of 106 HPs in *Salmonella* sp., 11 of which were identified as specific drug targets. Other studies analyzed the role of HPs in *Helicobacter pylori* using protein interaction networks and found significant involvement of HPs in flagellar assembly, bacterial chemotaxis, and lipopolysaccharide biosynthesis (34). In this study, we observed that the expression of two hypothetical protein genes significantly decreased after the addition of the antibacterial aptamer, suggesting that the two HPs play an important role in the inhibition of the aptamer. However, their specific functions are still unclear and need to be verified by further studies.

In contrast to traditional techniques, transcriptomics has provided a better insight into the pathogenicity of *P. plecoglossicida*. Shi et al. (35) generated a strain of *P. plecoglossicida* deficient in the fliL gene. Based on the transcriptome analysis, they found 126 differentially expressed genes when compared to the wild-type strain, 114 of which were down-regulated and 12 up-regulated. Sun et al. (36) analyzed the interaction between *P. plecoglossicida* and the host large yellow croaker using transcriptomics and found 81 significantly differentially expressed genes in *P. plecoglossicida* after the fliA gene was silenced, 77 of which were down-regulated and 4 up-regulated. These studies show that knockout or silencing of relevant genes in *P. plecoglossicida* leads to significant overall changes in the transcriptome, affecting 80–126 differentially expressed genes. Transcriptomic studies in other species of pathogenic bacteria show similar significant changes in differentia gene expression such as the inhibitory effects of *Rhizoma coptidis* and *Fructus schisandrae* on *Vibrio alginolyticus* (1,737 and 1,068 DEGs) (37, 38) and the inhibitory effect of a natural product, fingered citron essential oil (FCEO), on *Listeria monocytogenes* (1,553 DEGs) (39). These results indicate that the effects of these herbal medicines and natural products on the pathogens are broad and multi-targeted, and have a great impact on the gene expression of the target bacteria. However, our study showed that only seven differentially expressed genes were associated with the inhibitory effect of antibacterial aptamers. The number of identified genes was significantly lower than in the above studies, suggesting that the antibacterial aptamers may only act on a few target sites of *P. plecoglossicida* and have strong specificity. There are several possible reasons why antibacterial aptamers have such specific effects on gene expression in target bacteria. First, aptamer is a large molecule, which is not easy to enter cell. The aptamer probably binds to a membrane protein on surface of the bacteriaum at first, and then enters the bacterial interior through endocytosis (40). After entering, the aptamer also faces the risk of degradation by nucleases in the cell, so its amount that can play a role in the cell is relatively small. Second, aptamer is very specific, and can only act with some specific target molecules in the cell, or act with some specific nucleic acid sequences through base complementary pairing. However, small molecules such as traditional Chinese medicine are easy to enter cells, have diverse components, and act on many target molecules and target sites, so there are many differentially expressed genes. In addition, aptamer only affects some genes and some target molecules for a short period of time, and gene knockout is a permanent knockout of genes formed by long-term evolution of bacteria, and the impact on bacteria is permanently destructive, so it will affect the expression of many genes in bacteria. The outcomes of this research provide new possibilities for the development of targeted antibacterial drugs in the future.

## Conclusion

The transcriptome analysis of *P. plecoglossicida* in the presence of the antibacterial aptamer B4 identified seven differentially expressed genes, of which, the *sad* gene of the aldehyde dehydrogenase family proteins and the *metN1* gene of the ABC proteins were up-regulated. The *paaB* gene of the B subunit of phenylacetyl-CoA cyclooxidase, the two IS256 transposase genes, and two hypothetical protein genes with unknown functions were down-regulated. GO enrichment analysis of the differentially expressed genes showed that these genes were mainly involved in DNA-mediated translocation, phenylacetic acid catabolism, growth hormone catabolism, polyamine transport ATPase activity, betaine aldehyde dehydrogenase activity, and ABC transporter protein complexes. KEGG enrichment analysis of the differentially expressed genes showed that *sad* and *paaB* genes played major roles, and these genes were mainly involved in the metabolism of NADH, alanine, aspartate, glutamate, phenylalanine, and methyl butyrate, and microbial metabolism in different environments. The inhibitory effect of aptamers on *P. plecoglossicida* may be realized mainly through the influence of these differentially expressed genes by impacting the synthesis of important nucleic acids and proteins and influencing other related metabolic processes. Thus, our findings demonstrate the specificity of aptamers in selectively targeting bacteria with a set of genes. This study also provides a proof of concept for the potential application of aptamers to replace antibiotics in certain fields, thus minimizing off-target effects and fitness costs imposed on fish. Future investigations should explore the impact of aptamers on the gut microbiome of fish before proceeding with field applications.

## Data Availability

The datasets presented in this study can be found in online repositories. The names of the repository/repositories and accession number(s) can be found at: https://ngdc.cncb.ac.cn/gas/, CRA019590.

## Footnotes

^1^ https://ngdc.cncb.ac.cn/gsa/
